# Case Report: The Complexities of Managing Medications and the Importance of Deprescribing Anticholinergics in Older Adults

**DOI:** 10.3389/fphar.2021.584667

**Published:** 2021-04-16

**Authors:** Taylor Elliott, Lynne Eckmann, Daniela C. Moga

**Affiliations:** ^1^ Department of Pharmacy Practice and Science, College of Pharmacy, University of Kentucky, Lexington, KY, United States; ^2^ PRO2RX LLC Pharmacy Consulting Services, Lexington, KY, United States; ^3^ Department of Epidemiology, College of Public Health, University of Kentucky, Lexington, KY, United States; ^4^ Sanders-Brown Center on Aging, University of Kentucky, Lexington, KY, United States

**Keywords:** case report, anticholinergics, deprescribing, polypharmacy, medications

## Abstract

Potentially inappropriate anticholinergic medications (including over-the-counter products), polypharmacy, and the existence of communication barriers among members of the interprofessional team frequently contribute to clinical complexity in older adults. We present the case of a frail 86-year old female from the perspective of a community pharmacist managing outpatient medications and transitions of care. CD’s past medical history is significant for dementia, multiple falls, recurrent urinary tract infections, depression, cardiac arrhythmia, macular degeneration, chronic pain, depression, and cerebrovascular disease.

## Introduction

CD, the patient discussed in this case report, is a frail 86-year-old female with diagnosed dementia taking several medications to address multiple chronic conditions. CD’s past medical history is significant for dementia, multiple falls, recurrent urinary tract infections (UTIs), depression, cardiac arrhythmia, macular degeneration, chronic pain, depression, and cerebrovascular disease. Despite participating in a medication management program with access to a board certified geriatric pharmacist available for consultation at all times, as well as having around-the-clock caregivers and a specialized team of health care providers, this patient's trajectory illustrates the complexities of managing medications and transitions of care in older adults with dementia, as well as the importance of carefully assessing and deprescribing medications with anticholinergic properties.

CD enrolled in a medication management program on 03/27/2020. This program is an innovative community program in Kentucky providing pharmacy services that promote and prolong independent living, as well as control ongoing prescription costs. The goals of the program are to decrease the number of unwanted effects from medications in order to prevent falls, fractures, and other adverse events that may lead to loss of independence. As a note, while some insurance plans include basic medication reviews, the comprehensive services included in this program are not covered by Medicare or other insurance. Upon enrollment, the patient's entire medication regimen - including over-the-counter medications, vitamins, and herbal supplements-is analyzed to identify potentially inappropriate medications (e.g., anticholinergics and benzodiazepines) and drug-drug interactions. Prescribers (i.e., primary care providers and specialists treating chronic disease states) are contacted at enrollment and as needed thereafter to recommend medication therapy changes. Each week, a seven-day supply of chronic medications (including over-the-counters and vitamins/herbal supplements) is prepared and delivered to the patient's residence. After the weekly visit, the pharmacist reviews the previous week's medication supply for adherence, and follow-up is made with family members, caregivers, and/or health care providers as appropriate. In addition, acute medication changes are immediately addressed, and same day reconciliations are made.

The goals of this case report are threefold: 1) to demonstrate the importance of deprescribing inappropriate anticholinergic medications, 2) to highlight the consequences of viewing over-the-counter medications as benign, and 3) to emphasize the critical need for adequate transitions of care and communication among all members of the interprofessional team.

## Case Description

The patient discussed in this case report is a frail 86-year-old female with a past medical history significant for dementia, multiple falls, recurrent urinary tract infections (UTIs), depression, cardiac arrhythmia, macular degeneration, chronic pain, depression, and cerebrovascular disease. CD lives independently at home along with her adult daughter who previously suffered a traumatic brain injury. CD was enrolled in a medication management program on 03/27/2020. At that time, the patient and her daughter had a caregiver assisting for 3 h every day. The caregiver ensured the appropriate medications were taken in the morning and organized the evening doses for self-administration. However, the patient was unable to safely self-administer medications and was therefore referred to the medication management program. Upon enrollment, an electronic medication box that rotates and alarms at set times was provided to help manage medications. CD had nine chronic medications at enrollment (Table 1), including one anticholinergic (loratadine). Although loratadine is an anticholinergic medication, it is considered a safer alternative when an antihistaminic is necessary to prescribe. None of CD’s initially reviewed medications were potentially inappropriate for her based on the 2019 Beers List (By the 2019 American Geriatrics Society Beers Criteria® Update Expert Panel, 2019). The timeline of events relevant for this case is described below, and a summary is included in the supplementary table (Supplementary Table S1).

• April 20, 2020: CD’s initially reported medication list remained unchanged until this time. The caregiver alerted the pharmacist that the patient was having trouble sleeping. Melatonin was changed from a quick-dissolve tablet to a capsule due to the patient’s inability to appropriately use medications by sublingual route.
• April 21, 2020: Upon scheduled delivery, the caregiver notified the pharmacist the patient had experienced a fall the weekend prior and injured her shoulder. The caregiver also mentioned a suspected UTI due to the patient having cloudy urine. The caregiver contacted the primary care provider (PCP) about the patient’s signs and symptoms. In order to limit exposure during the COVID-19 pandemic, the PCP requested a urine sample to be dropped off in place of a traditional appointment. The following day, the PCP confirmed the presence of a UTI and prescribed cephalexin 250 mg capsules by mouth three times daily for 7 days. The pharmacist simultaneously initiated a 30-days course of probiotics in an attempt to prevent a *Clostridium difficile* infection. In addition, the PCP placed CD’s donepezil on hold and expressed concern that the medication may be contributing to the patient’s falls and lack of appetite. At the time of the initial writing of this report, the cause of CD’s fall was unknown. Possible causes include drug-drug interactions due to polypharmacy (see Table 1), UTI, and decreased cognitive function.
• April 24, 2020: The caregiver notified the pharmacist that the acetaminophen ER 650 mg tablets and lidocaine patches were not sufficiently relieving the patient’s pain that resulted from her fall. On April 28, 2020, the patient’s PCP prescribed tramadol 50 mg tablets by mouth three times daily as needed. At this time, the patient transitioned to 24-h, around-the-clock care. The original caregiver extended her daytime hours, and additional help was hired for evening assistance.
• May 12, 2020: The caregiver notified the pharmacist about the patient’s unimproved state – she was still confused, lethargic, and had poor appetite. The pharmacist suggested UTI test strips to evaluate the potential of a refractory UTI from April 22, 2020.
• May 13, 2020: The test strips were delivered to the patient’s residence by the pharmacist. The patient tested positive for nitrites and leukocytes. The pharmacist suggested a urinalysis, and culture and sensitivity after learning of the positive result.
• May 14, 2020: The PCP prescribed sulfamethoxazole/trimethoprim 400/80 mg by mouth two times daily for 10 days and instructed the caregiver to re-check for infection three days after completing the course of antibiotics.
• May 18, 2020: Upon scheduled delivery, the pharmacist incidentally noticed four bottles of liquid diphenhydramine that were not supplied by the pharmacy or recorded on the patient’s initial medication list. The caregiver informed the pharmacist she had seen it advertised for sleep. According to the caregiver, the patient had been taking 50 mg by mouth nightly for approximately one week. However, the exact initiation date remains unclear, as the diphenhydramine was not cleared by the pharmacist prior to use. The pharmacist discussed the potential adverse effects of anticholinergic medications in older adults with dementia (including increased risks for falls, urinary retention, and confusion) and suggested tapering the diphenhydramine to 25 mg nightly for a short period followed by discontinuation. Diphenhydramine is on the 2019 Beers List as a potentially inappropriate medication in due to its highly anticholinergic properties (Salahudeen et al., 2015; By the 2019 American Geriatrics Society Beers Criteria® Update Expert Panel, 2019).
• May 21, 2020: The patient was prescribed megestrol 400 mg/10 ml by mouth daily for appetite stimulation, along with a refill for tramadol. The prescriptions were sent in by a palliative care provider. Of note, a prior authorization was required for the megestrol, so the patient did not receive the medication prior to hospitalization of May 27, 20 (see below). In addition, the pharmacist had concerns that another medication was being added to CD’s already complex regimen without trying to find the root cause of her symptoms. In addition, megestrol is listed on the 2019 Beers List for increased risk of thrombotic events and possibly death in older adults.
• May 22, 2020: The caregiver alerted the pharmacist of the patient’s consistently low blood pressure (∼90/60 mmHg). The nurse practitioner on the palliative care team instructed the patient’s metoprolol be held. Upon review of the patient’s medication list, the pharmacist also suggested holding lisinopril and contacting the provider to further evaluate. At this time, the caregiver confirmed diphenhydramine was tapered and discontinued.
• May 27, 2020: CD’s conditioned continued to decline, and she was admitted to the hospital with a diagnosis of sepsis secondary to Gram-negative UTI. She also had hypokalemia. CD was treated with intravenous antibiotics while inpatient.
• June 01, 2020: CD was discharged from the hospital at 10:00 pm with hospice referral. The patient was sent home with three prescriptions (a new order for potassium and decreased doses of metoprolol and lisinopril) from the hospital’s “Meds to Beds” service.
• June 02, 2020: The pharmacist contacted the caregiver to check in and was informed of the patient’s discharge the night before. The pharmacist then attempted to contact the hospice nurse twice but did not receive a call back until after the home visit was already completed. Upon delivery, the pharmacist found handwritten notes from the hospice nurse on the medication list provided by the medication management program. The pharmacist reconciled the notes with the discharge paperwork from the hospital to ensure accuracy.
• June 08, 2020: lisinopril, metoprolol, and pravastatin were all discontinued per hospice. Per the pharmacist’s recommendations, CD’s doses of Eliquis were spaced to 9:00 am and 9:00 pm (rather than 9:00 am and 6:00 pm) and the oral potassium was changed to 20 mEq twice daily rather than 40 mEq once daily to reduce GI upset (Table 2).

**TABLE 1:** Initial Medication List (as reported on March 30, 2020)

Patient: CD (XX/XX/1934)	Initial Medication List (as reported on March 30, 2020)				
**Medication**	**Dose**	**9 AM**	Noon	6 PM	9 PM
Lisinopril 5 mg	1 daily	1			
Apixaban 2.5 mg	1 twice daily	1		1	
Metoprolol succinate 50 mg	1 daily	1			
Donepezil 5 mg	1 daily	1			
Simethicone 80 mg	Chew 1 three times daily	1		1	1
Loratadine 10 mg^a^	1 daily			1	
Preservision	2 daily			2	
Trazodone 50 mg	2 daily				2
Melatonin 10 mg	1 daily at bedtime				1
**Potential Drug-Drug Interaction**		**Risk Rating**	**Summary of Interaction**		
Donepezil (Acetylcholinesterase Inhibitors) ‐ Loratadine (Anticholinergic Agents)		C	Acetylcholinesterase Inhibitors may diminish the therapeutic effect of Anticholinergic Agents. Anticholinergic Agents may diminish the therapeutic effect of Acetylcholinesterase Inhibitors.		
Donepezil (Acetylcholinesterase Inhibitors) ‐ Metoprolol (Beta-Blockers)		C	Acetylcholinesterase Inhibitors may enhance the bradycardic effect of Beta-Blockers.		
Donepezil (Bradycardia-Causing Agents) ‐ Metoprolol (Bradycardia-Causing Agents)		C	Bradycardia-Causing Agents may enhance the bradycardic effect of other Bradycardia-Causing Agents.		
Loratadine (CNS Depressants) ‐ Trazodone (CNS Depressants)		C	CNS Depressants may enhance the adverse/toxic effect of other CNS Depressants.		

^a^ = anticholinergic properties.

Risk Ratings: A = No known interaction, B = No action needed, C = Monitor therapy, D = Consider therapy modification, X = Avoid combination.

**TABLE 2:** Updated Medication List (as reported on June 8, 2020).

Patient: CD (XX/XX/1934)	Updated Medication List (as reported on June 8, 2020)				
Medication	Dose	9 AM	Noon	6 PM	9 PM
Eliquis 2.5 mg	1 twice daily	1			1
**Potassium Cl 20 mEq**	**1 twice daily with food and water**	**1**			**1**
Loratadine 10 mg^a^	1 daily				
Preservision	2 daily			2	
Trazodone 50 mg	2 daily				2
Melatonin 10 mg	Dissolve 1 tablet at bedtime				1
**Trubiotic**	**1 daily for 60 d**			**0 or 1**	
**Tramadol^b^ 50 mg**	**1 three times daily as needed for pain**				
**Potential Drug-Drug Interaction**		**Risk Rating**	**Summary of Interaction**		
Loratadine (CNS Depressants) ‐ Potassium Chloride		X^c^	Anticholinergic Agents may enhance the ulcerogenic effect of Potassium Chloride.		
Loratadine (CNS Depressants) ‐ Tramadol (Opioid Agonists)		D^d^	CNS Depressants may enhance the CNS depressant effect of Opioid Agonists.		
Tramadol (Opioid Agonists) ‐ Trazodone (CNS Depressants)		D^d^	CNS Depressants may enhance the CNS depressant effect of Opioid Agonists.		
Tramadol (Serotonergic Opioids (High Risk)) ‐ Trazodone (Serotonergic Non-Opioid CNS Depressants)		D^d^	Serotonergic Non-Opioid CNS Depressants may enhance the CNS depressant effect of Serotonergic Opioids (High Risk). Serotenergic Non-Opioid CNS Depressants may enhance the serotonergic effect of Serotonergic Opioids (High Risk). This could result in serotonin syndrome.		
Loratadine (CNS Depressants) ‐ Trazodone (CNS Depressants)		C	CNS Depressants may enhance the adverse/toxic effect of other CNS depressants.		

Bolded = new,

^a^ = anticholinergic properties,

^b^ = on the Beers 2019 list.

^c^ Agents with greater anticholinergic effects are likely of more concern than agents with lesser anticholinergic effects like loratadine.

^d^ CD uses tramadol very infrequently and only as needed.

Risk Ratings: A = No known interaction, B = No action needed, C = Monitor therapy, D = Consider therapy modification, X = Avoid combination.

At the time this case report was written in July 2020, the pharmacist had suggested multiple medication changes to the hospice team and had also requested a BMP to check CD’s potassium level. She had not yet heard back. When revisions were made in February 2021, CD’s clinical status had improved significantly. The pharmacist’s recommendations where accepted (including the addition of potassium), the patient no longer required hospice services, and memantine was initiated to slow further cognitive decline.

## Discussion

**FIGURE 1:**
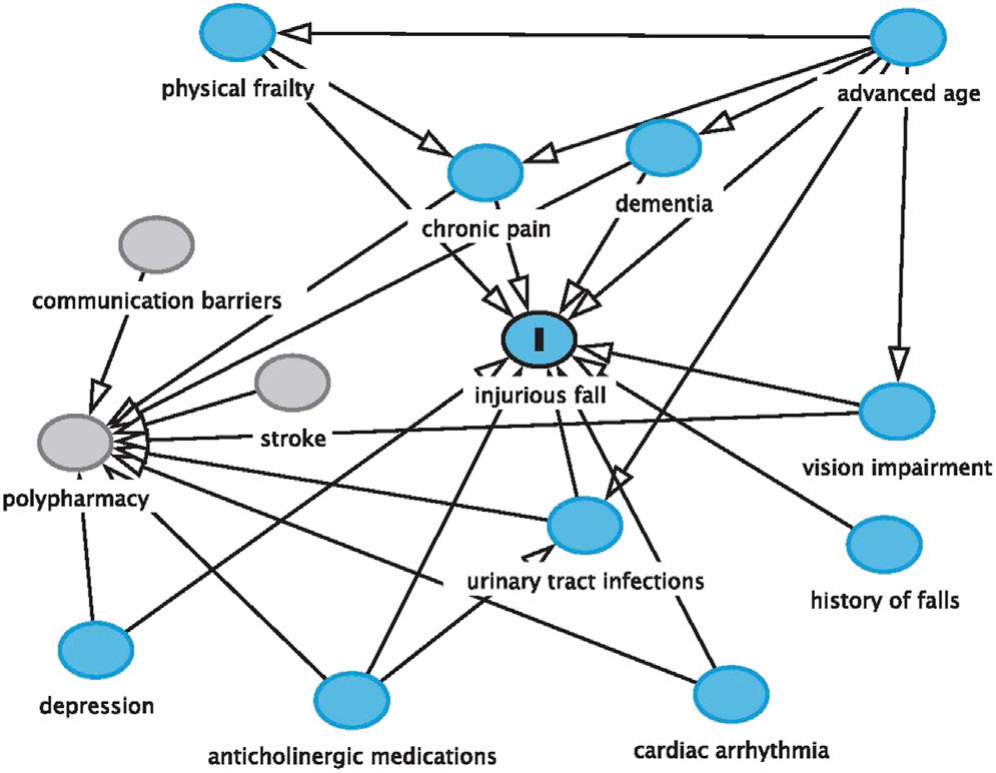
Factors associated with the risk of falls.

The patient discussed in this case report illustrates the complexities of managing medications in older adults with dementia. The potentially harmful use of an anticholinergic over-the-counter medication, the presence of polypharmacy, the patient’s impaired cognitive function, the manifestation of UTI, and the incidence and risk of recurrent injurious falls are all interconnected. Communication barriers among members of the interprofessional team and the global COVID-19 pandemic further complicated CD’s clinical course (Figure 1).

Anticholinergics are listed on the American Geriatrics Society’s 2019 Beers List as potentially inappropriate medications for older adults (By the 2019 American Geriatrics Society Beers Criteria® Update Expert Panel, 2019). Even as single agents, they may contribute to negative events such as falls, delirium, urinary retention, and decreased cognitive function. Despite these risks, as many as one in every two older Americans takes an anticholinergic medication. Because over-the-counter medication use is not easily captured in healthcare data, it is likely this statistic is underestimated. Non-prescription medications like diphenhydramine, doxylamine, and dimenhydrinate are commonly purchased and chronically used to treat conditions such as allergies, sleeplessness, and motion sickness (Holden et al., 2019). In fact, one study showed that more than half of the 1,025 participants aged 65 and older who were interviewed reported using a potentially inappropriate over-the-counter medication containing diphenhydramine or doxylamine to improve sleep within the past 30 days. Those participants were also less likely to be aware of the associated safety risks (Sudershan and Kamath, 2017).

Because over-the-counter medications like diphenhydramine and doxylamine are so easily accessible, they are often perceived as benign. Viewing them as low risk, patients and their caregivers frequently fail to report their use, as in the case of CD. This issue can go unnoticed for long periods of time and lead to new health problems or perpetuate existing issues. CD’s nightly use of diphenhydramine potentially contributed to her complicated clinical course through multiple mechanisms. First, as a member of the anticholinergic class, diphenhydramine independently increases fall risk. Because the exact initiation date remains unclear, it is difficult to determine whether the diphenhydramine contributed to the injurious fall that occurred the weekend prior to April 21, 2020. Given the widespread availability of these agents, it is possible they may have contributed to CD’s previous falls, and without pharmacist intervention, they certainly would have contributed to CD’s risk for recurrent falls. Secondly, because diphenhydramine leads to urinary retention, it may have promoted the growth of unwanted bacteria that are normally flushed out during micturition. Subsequently, the manifestation of a urinary tract infection may have led to increased confusion and/or low blood pressure and further increased fall risk as a result. Third, anticholinergic medications like diphenhydramine have been shown to exacerbate pre-existing cognitive impairment, as they counteract the therapeutic effect of medications used to treat dementia and other similar conditions. In CD’s case, the diphenhydramine was likely negating the potential benefit of donepezil. Not surprisingly, dementia is also considered an independent risk factor for falling in older adults.

While the use of a potentially harmful over-the-counter anticholinergic medication potentially contributed to CD’s clinical issues, it was not the only risk factor. Polypharmacy, which is commonly defined by the use of five or more medications daily, leads to an array of unwanted consequences: increased healthcare costs, adverse drug events, drug-drug and drug-disease interactions, medication non-adherence, decreased functional status, increased cognitive impairment, increased falls (and therefore increased morbidity and mortality), increased urinary incontinence, and increased incidence of malnourishment (Maher et al., 2013). Upon enrollment in the medication management program, the patient reported taking nine medications per day with three different dosing times. While each of these medications may have been indicated for a specific disease state, the risk of harm and adverse events has been shown to increase with each added medication (Rudolph, 2008; Moga et al., 2019).

Lastly, the lack of adequate transitions of care and communication among all members of the interprofessional team likely also contributed to the patient’s clinical course. Within the first three months of CD’s enrollment in the medication management program, she received care from her PCP, the palliative care team, the inpatient team during her hospital stay, the pharmacist, and her caregivers. Communicating the necessary information in a timely fashion to all participating healthcare providers proves itself nearly impossible, especially considering the lack of interfacing between electronic health record (EHR) systems. In addition, the global COVID-19 pandemic increased these difficulties, as CD was unable to seek traditional face-to-face outpatient care.

Unfortunately, the use of potentially inappropriate anticholinergic medications (including over-the-counter products), polypharmacy, and the lack of communication among the interprofessional healthcare team are widespread issues not limited to this patient. Bringing awareness to these problems is a crucial first step in improving health outcomes and prolonging independent living among older adults. Pharmacists providing medication management programs can play a key role as mediators between providers and patients and their caregivers in order to improve quality of care. As medication experts, pharmacists can provide medication therapy recommendations to prescribers, as well as provide appropriate education to patients and their caregivers. In the case of CD, the pharmacist’s continuity of care led to the discontinuation of diphenhydramine and potentially prevented further adverse events, including additional falls.

### Strengths

This case report serves as an educational tool for health care professionals managing the care of similar patient populations. It will hopefully encourage changes in clinical and community practice, including more deprescribing of potentially inappropriate medications, like anticholinergics and more vigilance during transitions of care.

### Limitations

One of the main limitations of this case report was the pharmacist’s lack of access to CD’s EHR. The inability to view patient care notes and lab results made it difficult to see the complete medical picture. However, this highlights the current reality of community pharmacists and demonstrates the need for improvement. Another limitation of this case report is that it is not generalizable to patients who aren’t enrolled in a medication management program. Older adults attempting to self-manage their medications are at even greater risk for adverse effects of medications.

## Patient Perspective

Note: Due to CD’s cognitive impairment, we requested the caregiver’s perspective in lieu of the patient perspective.

I had previously been a caregiver for the client for a short time after she returned home from hiatal hernia surgery in July 2019. In March 2020, I came back into her home after another caregiver left. Caregiving hours were set for 3 h daily. Shortly after coming back, the global COVID-19 pandemic really became something scary in the United States. I was relieved to have medication management support added by the client’s POA due to caregiver reported concerns of patient’s inability to self-manage and patient’s daughter mixing up medication planners during unsupervized times of the day. After the client’s daughter reported a fall, I reached out to the doctor. I was instructed to treat soreness with acetaminophen and monitor since no major injuries were observed. After coming back from being off over the weekend, patient’s daughter reported three falls. She also stated the ambulance came, but her mother refused to go the hospital. I believe she was scared of COVID-19. The next day, I called her physician to report the fall and right shoulder pain. We were able to set up a telehealth appointment since I could assist using my smartphone. Her doctor did not want to prescribe pain medication because of risk of falls and adverse effects and previous history. He did make a referral for palliative care evaluation, but this took weeks. I also reported concerns of increased confusion and hallucinations and questioned history of urinary tract infections. I was instructed to pick up sterile urine cups at the office and return sample. I did not hear back from the office, but I was informed by the pharmacist when an antibiotic had been prescribed and medication planner was updated. Unfortunately, the client did not seem to be improving. After another call to the doctor, due to client’s continued shoulder pain, the doctor prescribed tramadol. During scheduled weekly delivery, the pharmacist discovered over-the-counter diphenhydramine liquid had been added to help with sleep and explained this could worsen confusion, increase risk of falls, and cause urinary retention and should be discontinued. I appreciated the information, as I did not realize something promoted over the counter for sleep could cause these problems. The pharmacist suggested and delivered over the counter strips to see if infection was still there and recommended follow up with physician if positive. I called the office since the test showed positive, and the doctor called in a different antibiotic and asked me to check urine again with the strips after she finished. Unfortunately, the client continued declining and was admitted to the hospital with sepsis and low potassium. Since the client has come back home, hospice is in place and we continue to work through these challenges.

Three concerns I noted during the experience include: difficulty navigating the health system with multiple phone calls and delayed follow up return calls, complex situation complicated by COVID-19 and offices shifting to telehealth, and communication challenges due to multiple factors.

## Future Directions

Pharmacists, particularly those leading comprehensive medication management programs, are uniquely positioned to help incorporate non-pharmacologic fall risk reduction strategies. For example, patients like CD could undoubtedly benefit from physical therapists to improve gait and stability, nutritionists to enhance diet (i.e., calcium intake), and optometrists to assist with vision impairment. Serving as the patient’s liaison to health care providers of this sort could undoubtedly improve health outcomes and quality of life. In addition, pharmacists can help reduce environmental risk factors for falls by suggesting sensible shoes, increased lighting in dimly lit areas, assistive devices where appropriate, and removal of tripping hazards such as throw rugs.

## Data Availability

The original contributions presented in the study are included in the article/Supplementary Material, further inquiries can be directed to the corresponding author.
